# Molecular signatures of chronic myeloid leukemia stem cells

**DOI:** 10.1186/2050-7771-1-21

**Published:** 2013-06-06

**Authors:** Yaoyu Chen, Shaoguang Li

**Affiliations:** 1 Department of Oncology, Novartis Institutes for Biomedical Research, 250 Mass Ave, Cambridge, MA 02139, USA; 2 Department of Medicine, Division of Hematology/Oncology, University of Massachusetts Medical School, 364 Plantation Street, Worcester, MA 01605, USA; 3 Department of Medicine, University of Massachusetts Medical School, 364 Plantation Street, Worcester, MA 01605, USA

**Keywords:** BCR-ABL, Leukemic stem cells, CML, Biomarker, Hematopoietic stem cells, Cancer stem cells

## Abstract

*BCR-ABL* tyrosine kinase inhibitors (TKIs) are effective in controlling Philadelphia-positive (Ph^+^) chronic myeloid leukemia (CML) are unlikely to cure the disease because TKIs are unable to eradicate leukemia stem cells (LSCs) responsible for the disease relapse even after tyrosine kinase inhibition. In addition, the TKI resistance of LSCs is not associated with the *BCR-ABL* kinase domain mutations. These observations indicate that TKI-insensitive LSCs and TKI-sensitive leukemic progenitor cells are biologically different, which leads us to believe that LSCs and more differentiated leukemic cells have different genetic mechanisms. Further study of LSCs to identify the novel gene signatures and mechanisms that control the function and molecular phenotype of LSCs is critical. In this mini-review, we will discuss our current understanding of the biology of LSCs and novel genes that could serve as a molecular signature of LSCs in CML. These novel genes could also serve as potential targets for eradicating LSCs in CML.

## Introduction

Human Philadelphia chromosome-positive (Ph^+^) leukemia induced by the *BCR-ABL* oncogene is the most common myeloproliferative disorder known as chronic myeloid leukemia (CML). CML often starts with a chronic phase, which is characterized by granulocytosis and splenomegaly. The disease can progress to acute leukemia. CML starts with a chronic phase and progresses to a more acute terminal phase called “blast crisis” resulting in development of acute myeloid or acute B-lymphoid leukemia, which is characterized by granulocytosis and splenomegaly. More than ten years ago, a BCR-ABL kinase inhibitor called imatinib mesylate (Gleevec/Glivec, formerly STI571; Novartis) was approved by FDA for treating CML patients [1,2]. The rate of complete cytogenetic response among patients receiving imatinib was 87% after 5 years of treatment [3]. Although it effectively inhibits the BCR-ABL kinase activity and improves the survival of CML patients, imatinib does not appear to lead to a cure of the disease, because patients in complete cytogenetic remission after imatinib treatment still contain *BCR-ABL*-expressing leukemia cells. One explanation is that survival of those primitive CML cells were not dependent on BCR-ABL kinase activity so that therapies that biochemically target BCR-ABL could not eliminate CML stem cells [4,5]. Those CML patients will most likely be required to take the drug for the rest of their lives [6]. The resistance of LSCs to kinase inhibitors suggests that *BCR-ABL* may activate some unique and unknown molecular signaling pathways through both kinase-dependent and kinase-independent mechanisms in LSCs [7].

### *BCR-ABL*-expressing hematopoietic stem cells function as LSCs of CML and are resistant to BCR-ABL kinase inhibitors

Cancer stem cells (CSCs), including LSCs in CML, constitute a subpopulation of malignant cells capable of self-renewal and differentiation [8-12]. Recently, CSCs have been defined by their ability to repeatedly generate a continuously growing tumor [13]. Weissman and colleagues proposed that a candidate CSC population should exhibit the following properties: 1) The unique ability to engraft; 2) The ability to replicate the tumor of origin both morphologically and immunophenotypically in xenografts; and 3) The ability to be serially transplanted [13].

CML occurs because of clonal expansion of *BCR-ABL*-expressing hematopoietic stem cells. In CML patients, a *BCR-ABL* containing leukemic clone typically produces the myeloid lineage cells and B-lymphoid cells. LSCs in CML have some characteristics of normal hematopoietic stem cells. The *BCR-ABL* retroviral bone marrow transduction/transplantation mouse model has been widely used to establish a more efficient CML mouse model for studying the biology of LSCs [14]. By using the CML mouse model, *BCR-ABL*-expressing Lin^-^c-Kit^+^Sca-1^+^ cells were shown to function as LSCs in chronic phase CML [15].

BCR-ABL kinase inhibitors: imatinib, dasatinib and nilotinib were developed to treat CML and imatinib now serves as the frontline therapy for the patients with chronic phase CML [16]. Even though it can control CML development effectively, imatinib does not appear to cure the disease. One possible reason is that LSCs are insensitive to kinase inhibitors. Imatinib kills almost all dividing cells; however, a significant population of viable CD34^+^ cells are unaffected by the treatment and are leukemic in nature [5]. The fact that imatinib could not target the quiescent *BCR-ABL*-expressing LSCs made it apparent that imatinib treatment alone could not cure CML [5,17]. Human CML stem cells do not depend on BCR-ABL kinase activity for survival and are thus not eliminated by imatinib therapy. Imatinib inhibited BCR-ABL kinase activity to the same degree in all stem (CD34^+^CD38^-^, CD133^+^) and progenitor (CD34^+^CD38^+^) cells and in quiescent and cycling progenitors from newly diagnosed CML patients. Although short-term *in vitro* imatinib treatment reduced the expansion of CML stem/progenitors, cytokine support permitted growth and survival in the absence of BCR-ABL kinase activity that was comparable to that of normal stem/progenitor counterparts. Primitive human CML cells are insensitive to imatinib treatment and therapies that biochemically target BCR-ABL kinase activity will not eliminate CML stem cells [18]. The minimal effect of BCR-ABL kinase inhibitor on LSCs was also observed in the CML mouse model [15]. Neither imatinib nor dasatinib show a complete eradication of *BCR-ABL*-expressing HSCs.

### Identification of novel gene signatures of LSCs in CML

**Figure 1:**
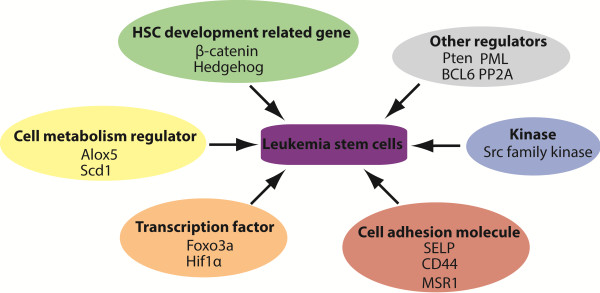
Different novel gene signatures regulate the function of LSCs.

Recently, several novel gene signatures in LSCs are identified to monitor the function and activity of LSCs after CML patients receive BCR-ABL kinase inhibitors treatment or other novel therapies (Table 1 and Figure 1).

**Table 1:** List of novel gene signatures in LSCs

**Gene name**	**Chr**	**Gene function**
β-catenin	Chr3	Cadherin-associated protein
Smo	Chr7	Smoothened, frizzled family receptor
Alox5	Chr10	Arachidonate 5-lipoxygenase
Scd1	Chr19	Stearoly-coenzyne A desaturase 1
Src kinase	Chr20	Kinase
Selp	Chr1	Granule membrane protein 140 kDa, antigen CD62
CD44	Chr11	Antigen
Msr1	Chr8	Macrophage scavenger receptor 1
Foxo3a	Chr6	Forkhead box O3
Hif1α	Chr14	Hypoxia inducible factor 1
Pten	Chr10	Prosphatase and tensin homolog
Bcl6	Chr3	B cell leukemia/lymkemia 6
PML	Chr15	Promyelocytic leukemia
PP2A	Chr19	Protein phosphatase 2A

#### *Cell metabolism regulators*

**Arachidonate 5-lipoxygenase** (*Alox5*) gene encoding arachidonate 5-lipoxygenase (5-LO) is involved in numerous physiological and pathological processes, including oxidative stress response, inflammation and cancer [19]. 5-LO is responsible for producing leukotrienes, such as LTB4, LTC4 and LTD4, a group of inflammatory substances that cause human asthma [19]. Altered arachidonate metabolism by leukocytes and platelets was reported in association with myeloproliferative disorders almost 30 years ago. Recently, *Alox5* was shown to be a critical regulator for LSCs in CML. Alox5 is significantly upregulated by BCR-ABL kinase and this upregulation does not depend on its kinase activity. In the absence of Alox5, *BCR-ABL* fails to induce CML in mice [20]. This Alox5 deficiency caused impairment of the function of LSCs but not normal hematopoietic stem cells (HSCs) through affecting differentiation, cell division and survival of long-term LSCs, consequently causing a depletion of LSCs and a failure of CML development. Similar results were obtained when mice with CML were treated with a 5-LO inhibitor. Human CML microarray studies also showed that *Alox5* is differentially expressed in CD34^+^ CML cells, suggesting a role of *Alox5* in human CML stem cells. This data suggests that Alox5 and its pathway plays an important role in self-renewal and differentiation of LSCs and could be potential biomarkers for monitoring the activity of LSCs in patients [20].

**Stearoyl-CoA desaturase 1** (Scd1) is an endoplasmic reticulum enzyme, belonging to a family of Δ9-fatty acid desaturase isoforms. Scd1 catalyzes the biosynthesis of monounsaturated fatty acids from saturated fatty acids, which are the most abundant fatty acids present in mammalian organisms [21]. The expression of the Scd1 gene is downregulated in LSCs and Scd1 plays a tumor-suppressive role in LSCs with no effect on the function of normal HSCs. Deletion of Scd1 causes acceleration of CML development and conversely overexpression of Scd1 delays CML development. In addition, Pten, p53, and Bcl2 are regulated by Scd1 in LSCs. Furthermore, the induction of Scd1 expression by a PPARγ agonist suppresses LSCs and delays CML development [22].

#### *Genes related to normal development of hematopoietic stem cells (HSCs)*

***β-catenin*** is a key factor in HSC development, and is activated by Wnt ligand binding to the receptor. Its stability after activation is highly regulated by a destruction complex involving the tumor suppressor Adenomatous Polyposis Coli (APC), the scaffolding protein that binds newly synthesized *β-catenin*. Two kinases Choline Kinase (CKI) and Glycogen Synthase Kinase 3β (GSK3β), phosphorylate Ser and Thr residues in the amino terminus of *β-catenin *[23]. In CML patients undergoing blast crisis, *β-catenin* is activated in myeloid progenitors and the activated *β-catenin* translocates to the nucleus [24]. In CML mouse model, deficient of *β-catenin* causes a reduced ability of *BCR-ABL* to support long-term renewal of LSCs, as shown in the serial replating and transplantation assays [25]. The inhibitory role of β-catenin in LSCs is associated with the decreased levels of p-Stat5α and associated with the overexpression of ABCB1, which gives rise to the multidrug resistance (MDR) phenomenon [25]. Antagonism of this pathway also led to impaired NFAT activity, decreased cytokine production, and enhanced sensitivity to BCR-ABL inhibition [26]. It is also known that increased exogenous Wnt-mediated β-catenin signaling played an important role in mesenchymal stromal cells -mediated protection of CML progenitors from tyrosine kinase inhibitor treatment [27]. Wnt/β-catenin signaling pathway was also shown to be required for the development of LSCs in AML as well as CML [28].

**Hedgehog** (Hh) pathway plays crucial role during embryonic development, tissue regeneration and repair in adults. Zhao et al. (2009) have demonstrated that loss of Smoothened (Smo), an important molecule of the Hh pathway, impairs HSC renewal and results in depletion of CML stem cells [29], although the effect of loss of Hh signaling through conditional deletion of Smo on adult hematopoiesis is still controversial [30,31]. The possible mechanism for the Smo action may be the upregulation of cell fate determinant Numb in the absence of Smo activity, which is responsible for depletion of CML stem cells [32]. Hh pathway activity is required for maintenance of normal and leukemia stem cells of the hematopoietic system.

#### *Kinases*

**Src family kinases (SFKs)** can be potential biomarkers for BCR-ABL kinase inhibitor resistant CML stem cells. SFKs were reported to be involved in tyrosine kinase inhibitor-resistant CML and elevated SFK activities were observed in patients with advanced disease in blast crisis. In a study of four categories of imatinib- and dasatinib-treated patients (imatinib resistant/dasatinib-responsive; dasatinib-resistant; blast crisis or CML progression and T315I or F317L mutated patient receiving omacetaxine treatment) [33], transcriptional and translational levels of HCK, LYN and another SFK-related gene BTK were elevated in more than 50% of resistant CML patients. This increase more significantly correlates with disease progression in a large population of CML patients. Interestingly, activation of SFKs expression is not likely caused by BCR-ABL mutation, as four BCR-ABL mutated patients showed concomitant SFK activation similar to that seen in patients expressing a wild-type BCR-ABL [33]. Recently, in BCR-ABL-induced chronic myeloid leukemia animal model, the *Blk* gene (encoding B-lymphoid kinase, a SRC family kinase) was shown to function as a tumor suppressor in LSCs but it did not affect normal HSCs or hematopoiesis. *Blk* suppressed LSC function through a pathway involving an upstream regulator, *Pax5*, and a downstream effector, *p27*. Inhibition of the *Blk* pathway accelerated CML development, whereas increased activity of the *Blk* pathway delayed CML development. *Blk* also suppressed the proliferation of human CML stem cells [22].

#### *Cell adhesion molecules*

Adhesion molecules such as **P-selectin** (encoded by the *Selp* gene) are essential for normal hematopoiesis, and their dysregulation has been linked to leukemogenesis [34-36]. Like HSCs, LSCs depend upon their microenvironments for survival and propagation. P-selectin plays a crucial role in Philadelphia chromosome-positive CML [34]. The cells deficient in P-selectin expression can repopulate the bone marrow more efficiently than wild type control cells [36]. This results from an increase in HSC self-renewal rather than alternative possibilities like increased homing velocity or cell cycle defects [36]. Recipients of *BCR-ABL*-transduced bone marrow cells from P-selectin-deficient donors develop more aggressive CML, with increased percentages of LSCs and progenitors [34]. Taken together, P-selectin expression on HSCs and LSCs may have important functional ramifications for both hematopoiesis and leukemogenesis, which is most likely attributable to an intrinsic effect on stem cell self-renewal [36].

Mouse *BCR-ABL*-expressing stem/progenitor cells express a higher level of **CD44**, which contributes functional E-selectin ligands [37,38]. In a mouse retroviral transplantation model of CML, *BCR-ABL*-transduced progenitors from CD44-mutant donors are defective in homing to recipient bone marrow, resulting in decreased engraftment and impaired induction of CML. By contrast, CD44-deficient stem cells transduced with empty retrovirus engraft as efficiently as do wild-type HSCs. CD44 is dispensable for induction of acute B-lymphoblastic leukemia by *BCR-ABL*, indicating that CD44 is specifically required for leukemic cells that initiate CML. The requirement for donor CD44 is bypassed by direct intrafemoral injection of *BCR-ABL*-transduced CD44-deficient stem cells or by coexpression of human CD44. Antibody to CD44 attenuates induction of CML in recipients. These results show that *BCR-ABL*-expressing leukemic stem cells depend to a greater extent on CD44 for homing and engraftment than do normal HSCs, and argue that CD44 blockade may be beneficial in autologous transplantation in CML [37]. Loss of CD44 was also reported to be able to alleviate the CML phenotypes in Kras G12D mice and attenuate aberrant GM-CSF signaling in Kras G12D cells [39].

Another cell adhesion molecule **MSR1** is a mutifunctional cell surface receptor and downregulated by *BCR-ABL*. This downregulation is partially restored by *Alox5* deletion, and that *Msr1* deletion causes acceleration of CML development [40,41]. Moreover, *Msr1* deletion markedly increases LSC function through its effects on cell cycle progression and apoptosis. MSR1 was also shown to affect CML development by regulating the PI3K-AKT pathway and β-Catenin, which suggesting that MSR1 suppresses LSCs and CML development [41]. The lower cell surface expression of MSR1 may also be used to monitor the activity of CML stem cells in patients.

#### *Transcription factor*

**Forkhead O transcription factor3a **(*Foxo3a*) was shown to play an essential role in the maintenance of LSCs of CML. Cells with nuclear localization of FOXO3a and decreased AKT phosphorylation are enriched in the LSC population. Serial transplantation of LSCs from *Foxo3a*^+/+^ and *Foxo3a*^-/-^ mice shows that the ability of LSCs to cause disease is significantly decreased by *Foxo3a* deletion. Furthermore, TGF-beta is also shown to be a critical regulator of AKT activation in LSCs and control FOXO3a localization. A combination of TGF-beta inhibition, *Foxo3a* deficiency and imatinib treatment led to efficient depletion of CML *in vivo *[42]. The inhibitory effect of FOXO3a on leukemia cells was shown through increasing PI3K/AKT activity in drug-resistant leukemic cells [43]. Recently, BCL6 proto-oncogene was also shown as a critical effector downstream of FoxO in self-renewal signaling of CML stem cells [43].

**Hypoxia-inducible factor-1α** (*Hif1α*) is a master transcriptional regulator of the cellular and systemic hypoxia response, and is essential for the maintenance of self-renewal capacity of normal HSCs and LSC of acute myeloid leukemia [44]. HIF1α also plays a crucial role in survival maintenance of LSCs of CML. Deletion of *Hif1α* impairs the propagation of CML through impairing cell-cycle progression and inducing apoptosis of LSCs. Deletion of *Hif1α* results in elevated expression of *p16* (*Ink4a*) and *p19* (*Arf*) in LSCs, and knockdown of *p16* (*Ink4a*) and *p19* (*Arf*) rescues the defective colony-forming ability of *Hif1α*^-/-^ LSCs. Compared with normal HSCs, LSCs appear to be more dependent on the HIF1α pathway [45].

#### *Other genes*

The tumor suppressor gene ***Pten ***is also downregulated by *BCR-ABL* in LSCs of CML mice. By genetic deletion or overexpression of *Pten*, it was shown to function as a tumor suppressor in LSCs of CML, consistent with the role of PTEN in LSCs of acute myeloid leukemia and progenitor cells of T-ALL progenitors. Functional enhancement of the PTEN pathway provides a therapeutic strategy for targeting LSCs [46].

***Bcl6 ***is a known proto-oncogene that is frequently translocated in diffuse large B cell lymphoma (DLBCL) [47]. In response to TKI-treatment, BCL6 protein level was upregulated by 90 folds in *BCR-ABL*-positive acute lymphoblastic leukemia cells. BCL6 upregulation upon TKI-treatment leads to transcriptional inactivation of P53 pathway and BCL6-deficient leukemia cells fail to inactivate P53 and are particularly sensitive to TKI-treatment. *Bcl6*^-/-^ leukemia cells are poised to undergo cellular senescence and fail to initiate leukemia in serial transplant recipients. A combination of TKI-treatment and a novel BCL6 peptide inhibitor markedly increased survival of NOD/SCID mice xenografted with patient-derived *BCR-ABL1* ALL cells [48].

**PML** functions as a tumor suppressor that controls fundamental processes such as apoptosis, cellular proliferation and senescence [49,50]. PML was revealed to have an indispensable role in maintaining LIC quiescence. *Pml-*deficient long-term LSCs become exhausted with time and are incapable of generating CML in transplanted animals [51].

**PP2A** is a phosphatase regulating many cellular functions and is genetically inactivated in many types of cancer [52]. PP2A activity is suppressed in blast crisis but not chronic phase CML cells through inhibition of BCR-ABL. Restoration of PP2A activity inhibits BCR-ABL expression and activity, hence impairing wild-type and T315I *BCR-ABL* leukemogenesis. In addition, pharmacologic enhancement of PP2A may represent a possible therapeutic strategy for blast crisis and imatinib-resistant CML [53].

## Summary

Although our current knowledge of the biology and therapy of CML LSCs is still limited, the identification of novel gene signatures, such as HSC development related genes, cell metabolism regulators, kinases, cell adhesion molecules and transcription factors (Table 1), provide the new opportunities for not only monitoring the proliferation of CML stem cells, but also developing promising anti-stem cell therapies for curing CML. Future clinical trials for testing those gene signatures in CML patients will determine whether novel *BCR-ABL* kinase inhibitors or other combinational therapies are effective in killing CML stem cells and curing the patients.

## Competing interests

The authors declare that they have no competing interests.

## Authors’ contributions

YC and SL wrote the manuscript. All authors read and approved the final manuscript.
